# Design and Fabrication of a Microfluidic Chip for Particle Size-Exclusion and Enrichment

**DOI:** 10.3390/mi12101218

**Published:** 2021-10-06

**Authors:** Luxia Yang, Tian Ye, Xiufeng Zhao, Taotao Hu, Yanlong Wei

**Affiliations:** Department of Computer, Taiyuan Normal University, Taiyuan 030619, China; masteryetian@163.com (T.Y.); zhaoxiufeng@tynu.edu.cn (X.Z.); Hutt@tynu.edu.cn (T.H.); 18636136726@163.com (Y.W.)

**Keywords:** microfluidic chip, 3D focusing channel, the micropillar array, electromagnetic drive, micro particles

## Abstract

Based on the size of particles, a microfluidic chip integrating micro particles capture, controlled release and counting analysis was designed and fabricated in this paper. The chip is composed of a polydimethylsiloxane (PDMS) cover sheet and a PDMS substrate. The PDMS substrate is made of a sample inlet, microfluidic channels, a micropillar array, a three-dimensional (3D) focusing channel, and a sample outlet. The chip was fabricated by the multistep SU-8 lithography and PDMS molding method in this study. The micropillar array and channels in the chip can be molded in one step and can be replicated multiple times, which reduces the production cost and increases the practicability of the chip. Using a homemade electromagnetic drive device, the detection function of the chip was tested using a deionized water solution containing 22 μm polyethylene particles. The results showed that under the action of electromagnetic force, the chip enriched polyethylene particles; when the electromagnetic force disappeared, the enriched polyethylene particles were released by injecting buffer solution, and it was looked at as new sample solution. The flow rate of the sample solution and the sheath flow solution (deionized water) was injected into the three-dimensional focusing channel at a flow rate ratio of 1:4, and the polyethylene particles sample solution was focused, which could be used for the counting and analysis of polyethylene particles. The work of this paper can provide a reference for the subsequent detection of circulating tumor cells (CTCs).

## 1. Introduction

Microfluidic chips, also known as labs-on-a-chip, are a technology integrating the functions of an entire analysis laboratory, including sampling, sample pretreatment, reaction, isolation, and detection, on a chip of a few square centimeters [1]. They work on the principles of biochemistry and analytical chemistry, and they often have the structural feature of a microchannel network. Microfluidic chips have the advantages of low reagent consumption, low sample quantity requirement, high functional integration, small chip size, and automatic operation. In recent years, with the continuous advancement of microfluidic chip technology, they have become a commonly used technology for precision diagnosis as part of precision medicine [2,3,4].

Circulating tumor cells (CTCs) are tumor cells that are shed from in situ tumor lesions and enter the peripheral blood circulation, which is a necessary prerequisite for tumor metastasis. However, the number of CTCs in peripheral blood is extremely low: In 10 mL of whole blood, there are approximately 80 billion red blood cells (RBCs), approximately 500 million white blood cells (WBCs), and only 1–100 CTCs [5], which poses a great challenge to the capture and counting technologies. Because the size of the microfluidic channel of the microfluidic chip is of the micrometer scale, and fluid at the micrometer scale has a unique laminar flow effect, which is conducive to the sorting of cells in the microfluid, this technology has great development potential and wide application prospects in the detection of CTCs. In recent years, microfluidic chips aimed at the sorting and enrichment of CTCs target cells have attracted the attention of many researchers [6,7,8,9,10], and such microfluidic chips have been designed and developed. 

The sorting and enrichment methods of these chips for CTCs are mainly divided into specific methods and nonspecific methods [11,12]. The former has complicated operating steps and limited flow rates, and their capture efficiency is easily affected by the contact area and contact probability between the internal structure of the chip and the cells. The latter has the advantages of a simplified operation process and high-throughput sorting ability, and the cells are not easily damaged during the process. Chung et al. [13] designed and studied a filter microfluidic chip with a dam structure. When sorting and enriching CTCs, the cells did not block the channel, and cell activity was not destroyed. Liu et al. [14] designed a triangular micropillar array in the microfluidic chip to achieve the sorting and enrichment of CTCs. Lv et al. [15] designed a microfluidic chip with a micropillar array at 8 μm spacing and applied it to the capture of MCF-7 breast cancer cells. These chips each have advantages in sorting speed, efficiency, purity, or cell viability, but they cannot perform on-chip detection and analysis of the captured CTCs. That is, the captured CTCs need to be transferred to a centrifuge tube for traditional detection and analysis using other equipment, which may cause CTC contamination or loss during the transfer process, resulting in low detection accuracy and low efficiency. 

This study puts forth a microfluidic chip structure design and manufacturing process that integrates CTC enrichment, sorting, and counting. The chip contains a micropillar array with 20 μm spacing and a three-dimensional (3D) focusing microfluidic channel. It realizes the enrichment, sorting, and counting of CTCs on a polydimethylsiloxane (PDMS) chip using a homemade electromagnetic drive device. This chip has a simple structure and low production cost and is ready for mass production. It is disposable to avoid cross-contamination, which improves detection accuracy and efficiency. It is expected to be applied to the detection of CTCs in the future.

## 2. Working Principle and Experimental Equipment

### 2.1. Enrichment and Sorting Principle of Specific Size Micro Particles by Microfluidic Chip

**Figure 1:**
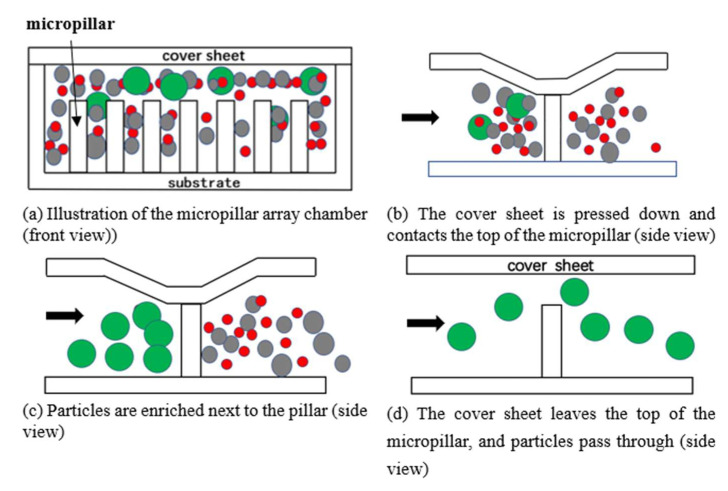
Schematic diagrams of the micro particles enrichment and sorting principles of the microfluidic chip.

The microfluidic chip for the enrichment, sorting, and counting consists of polydimethylsiloxane (PDMS) microfluidic channels, a micropillar array, and a PDMS cover sheet to form a closed cavity, as shown in Figure 1a. The core of the design is to integrate the enrichment and sorting structure and the 3D focusing microfluidic channel structure on the same chip. The former is for sample pretreatment of containing specific size particles to enrich micro particles, and the latter is designed based on our previous work [16,17] using a microfluidic cytometer to count the micro particles enriched.

The design principle of the enrichment and sorting structure is as follows. Based on the differences in physical size and deformability of micro particles, a micropillar array structure with a specific spacing size is incorporated into the microfluidic channel (the spacing size is smaller than the diameter of the micro particles), and the height of the micropillar array is smaller than the depth of the microfluidic channel (ensuring that the channel is unblocked when there is no external force). 

The microfluidic chip containing the above structures is placed on a homemade electromagnetic drive device, and the electromagnetic force generated by the electromagnetic drive device causes the PDMS cover sheet on top of the micropillar array to undergo downward elastic deformation, so that the top of the micropillar array contacts the upper surface of the cover sheet, as shown in Figure 1b. Because the spacing between the micropillars is smaller than the diameter of the micro particles and the micro particles are not easily deformed, the micro particles will be blocked at the space between the micropillars, and the unwanted sample particles will flow through normally. When the sample fluid is flowing, the micro particles will be enriched (blocked) in the vicinity of the micropillars, as shown in Figure 1c. When the electromagnetic force disappears, the cover sheet returns to its original position (Figure 1d), and the enriched micro particles can pass through the gap between the micropillar array and the cover sheet along with the flow of buffer, providing a sample with a high concentration for later counting and detection. 

### 2.2. Principal of the Micro Particles Counting

**Figure 2:**
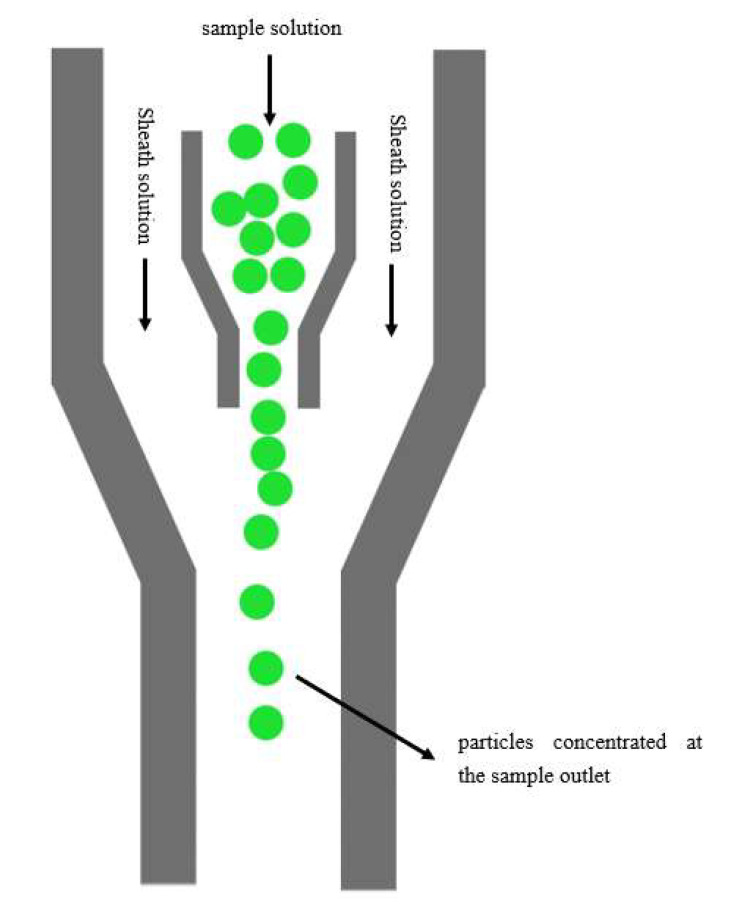
Principle diagram of microfluidic cytometer counting.

Based on the microfluidic cytometer [16,17] in our previous studies, three sample inlet channels were designed. The middle one is the micro particles-containing sample channel, and the two on both sides are sheath solution channels (the sheath solution is usually deionized (DI) water). The sheath solution and sample solution are injected into the corresponding channels at the same time using a microinjection pump. According to the principle of hydraulic focusing, the sample solution enclosed by the surrounding sheath solution can focus the sample solution in the middle of the focusing channel. By adjusting the flow-rate ratio of the sample solution to the sheath solution during injection, the focusing width of the sample solution can be narrowed down to allow only one row of micro particles in the sample solution to pass through the detection area. Light-emitting and receiving devices are installed on both sides of the detection area. When fluorescence-labeled micro particles pass through the detection area, they are excited by the excitation light to emit different fluorescence wavelengths. The micro particles are counted based on optical fiber collection data or image collection data as shown in Figure 2. 

### 2.3. Electromagnetic Drive Device Design

**Figure 3:**
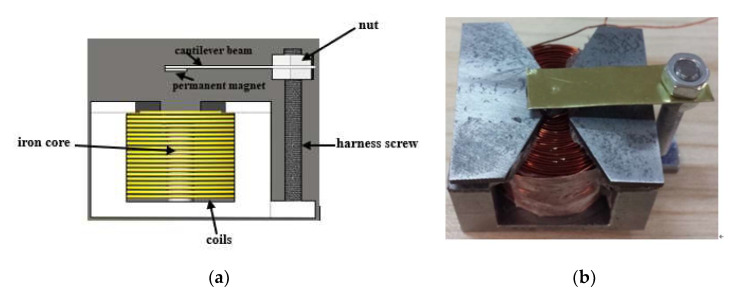
Diagram of the electromagnetic drive device. (**a**) Schematic diagram. (**b**) Picture of the actual device.

**Figure 4:**
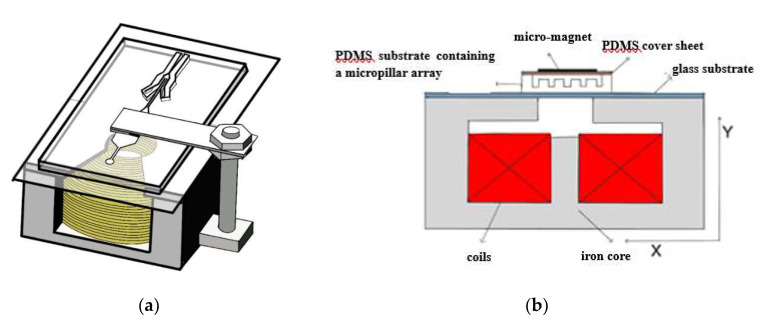
Diagram of the electromagnetic drive device with chip. (**a**) 3D diagram. (**b**) Front view diagram.

**Figure 5:**
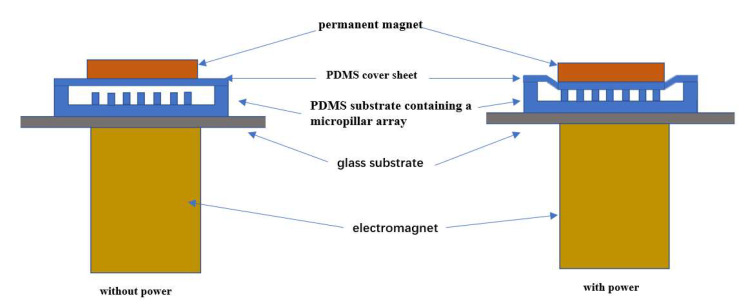
Diagram of the working principle of the electromagnetic drive device.

The homemade electromagnetic drive device for microfluidic chip is shown in Figure 3. It consists of coils, an iron core, harness screw, two nuts, a cantilever beam, and a permanent magnet. Figure 4 shows the 3D diagram and the front-view diagram when the chip is placed in the electromagnetic drive device. The basic working principle is as follows: without power, the cantilever beam and the permanent magnet are far away from the microfluidic chip that is located vertically below; with power, the electromagnetic coils generate an electromagnetic force, which attracts the permanent magnet on the cantilever beam, and the permanent magnet presses down the PDMS cover sheet, causing the cover sheet to deform and attach to the top of the micropillar array in the microfluidic channels to block the outflow of micro particles, achieving micro particles enrichment (Figure 5). We designed and fabricated an electromagnetic drive device meeting all the relevant requirements. The designed electromagnetic coils have an iron core with a diameter of 6 mm, 30 radial turns, 40 axial turns, and a total of 1200 turns. By adjusting the current, different electromagnetic forces can be generated. 

## 3. Design and Fabrication of the Microfluidic Chip

### 3.1. Force Analysis of the Microfluidic Chip

**Figure 6:**
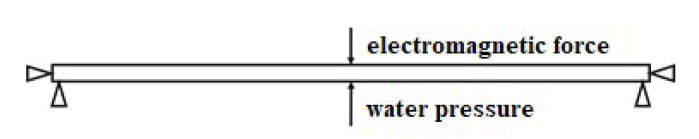
Simplified force mechanical model of the PDMS cover sheet.

As shown in Figure 5, the microfluidic chip is not subject to external force when the power is off, while with power, the cover sheet is subjected to external electromagnetic pressure and is elastically deformed to contact the top of the micropillar array. The width, length, and height of the micropillars of the micropillar array in the microfluidic channels are 250, 500, and 150 μm, respectively, and the depth of the microfluidic channel is 350 μm. When the PDMS cover sheet is put on the microfluidic channel substrate, the initial distance from the top of the micropillar array to the cover sheet is approximately 200 μm (obtained by subtracting the micropillar height from the channel depth). Therefore, to block micro particles in the sample solution, a 200 μm deformation of the PDMS cover sheet above the micropillar array structure must be caused by the electromagnetic drive device. The deformation model of the cover sheet under force is simplified into a mechanical model with two fixed ends and a force at the center. The force mainly comes from the downward electromagnetic force, as shown in Figure 6. 

For the analysis of deformation at any point of a film fixed at both ends under a uniform load, the formulas are as follows:

(1) $$\omega \left(x,\;y\right)=\frac{16q_{0}}{\pi^{6}D}\sum_{m=1,3,5,\ldots \ldots }^{\infty }\sum_{n=1,3,5,\ldots \ldots }^{\infty }\frac{\sin\frac{m\pi x}{a}\sin\frac{m\pi x}{b}}{mn(\frac{m^{2}}{a^{2}}+\frac{n^{2}}{b^{2}}{)}^{2}}$$

(2) $$D=\frac{Eh^{3}}{12(1-\mu^{2})}$$

where ω is the deformation of the film, Dis the bending stiffness of the square film, Eis the elastic modulus of the PDMS film, h is the thickness of the film, μ is the Poisson’s ratio of the PDMS film, x,y are the coordinates of any point on the film surface, $q_{0}$ is the distribution of the uniform load on the film, a is the length of the film, and b is the width of the film. In this study, the thickness of the cover sheet is 400 μm. The force area of the cover sheet is the same as the area of the micromagnet above it, which is a square of approximately 5 × 5 mm, ω = 200 μm, E = 0.5 MPa, and μ = 0 49. Substituting these known values into Formulas (1) and (2), the force required for the cover sheet deformation can be calculated in Matlab software. 

### 3.2. Fabrication of the Microfluidic Chip

**Figure 7:**
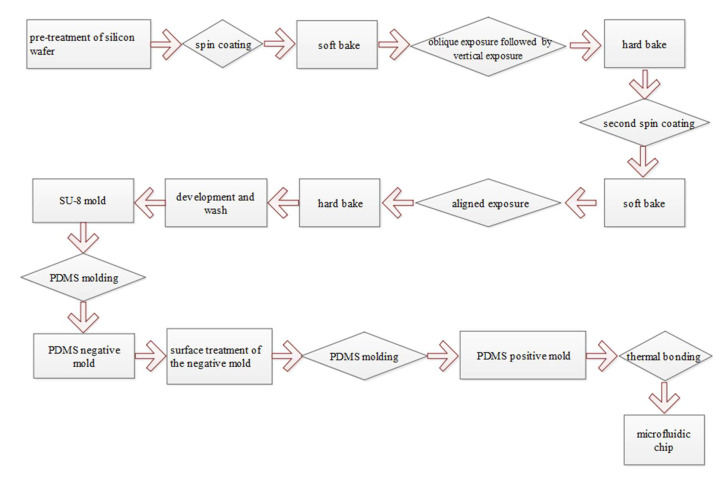
Process flowchart.

The microfluidic chip designed in this study is composed of a PDMS cover sheet and a PDMS substrate. The PDMS substrate is composed of a sample inlet, microfluidic channels, a micropillar array, a 3D focusing channel, and a sample outlet. The chip is fabricated using the micro-electromechanical-systems (MEMS) process. First a SU-8 mold is made, and then a PDMS replica is obtained using the PDMS dual-casting process. The specific workflow is shown in Figure 7. The surface-treated PDMS negative mold can be used as a mold for multiple casting to obtain the PDMS positive mold, realizing batch production of the chip. 

**Figure 8:**
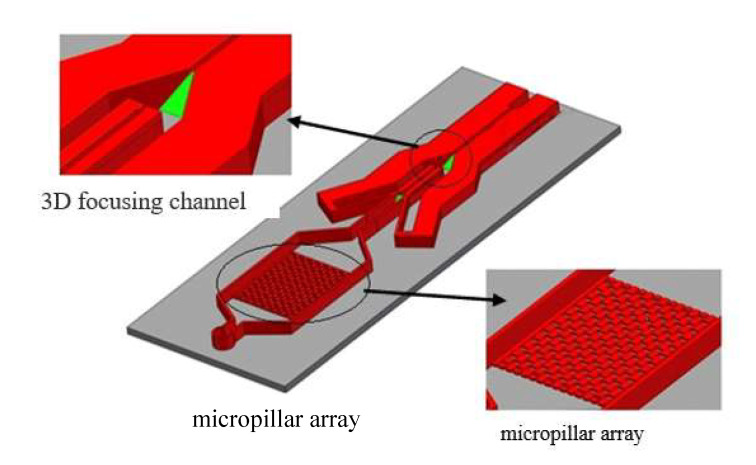
Structural diagram of the microfluidic chip.

Since the microfluidic chip integrates a micropillar array and a 3D focusing channel, the fabrication of the SU-8 mold is the key step of the entire process. At the same depth of the photoresist layer, the 3D focusing microfluidic channel is prepared by immersive oblique exposure, while the micropillar array requires vertical exposure, so the two parts cannot be formed at the same time. Therefore, we designed a two-layer overlay photomask for SU-8 lithography. First, oblique exposure was performed as described [17]. Next, the oblique exposure pattern was covered to perform vertical exposure for the micropillar array. A hard bake was performed at 65 °C for 20 min, and 95 °C for 1 h. The hard baked film was spin-coated with a second layer of SU-8 photoresist at a thickness of approximately 200 µm, which is the depth of the outlet channel. Finally, after vertically aligned exposure, hard baking, and development, an SU-8 mold with an integrated micropillar array and 3D microfluidic focusing channel was obtained. The specific structure of the chip is shown in Figure 8. 

Next, a PDMS prepolymer was coated onto the SU-8 mold. After removing bubbles in a vacuum box and heating in an oven to 80 °C for 1 h, the PDMS negative mold that was opposite to the SU-8 mold was obtained. The negative mold was surface-treated as described [16], and then a second PDMS prepolymer coating was performed. As described above, after heating and curing, the PDMS microfluidic channel structure was peeled off from the PDMS negative mold, which was the same as the original SU-8 mold and was the substrate of the microfluidic chip. Finally, the PDMS prepolymer and curing agent were mixed at a mass ratio of 15:1, stirred evenly, vacuumed, and coated on cleaned silicon wafer. The PDMS cover sheet was placed in a vacuum oven at 75 °C for 30 min, then slowly cooled to room temperature. The incompletely cured PDMS cover sheet was peeled off from the silicon wafer and was quickly bonded to the above PDMS substrate. After removing bubbles, the chip was placed in a vacuum drying oven at 65 °C overnight to form permanent bonding. 

## 4. Experiments and Testing Results

### 4.1. Chip Fabrication Results

**Figure 9:**
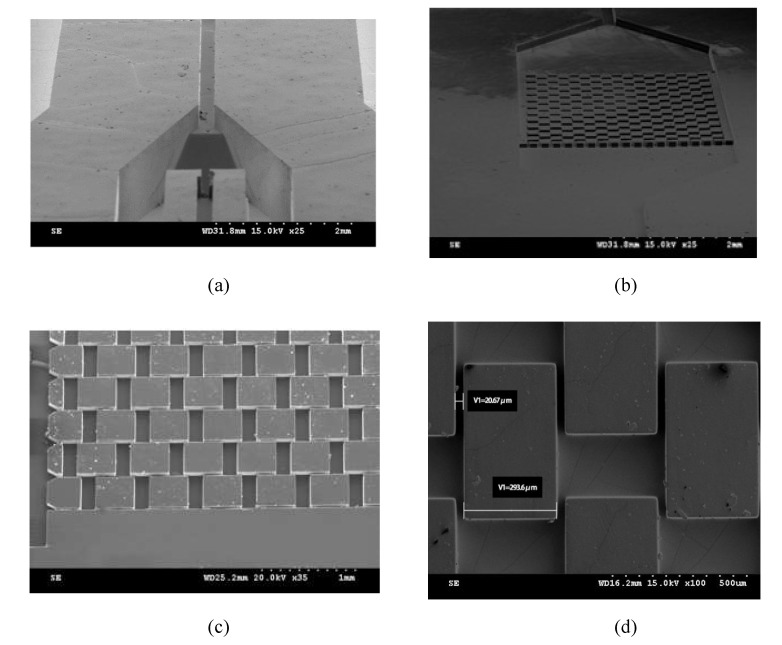
Scanning electron micrographs of parts of the PDMS microfluidic chip. (**a**) The slope structure of the microfluidic focusing channel. (**b**) Micropillar array structure. (**c**) Enlarged view of a part of the micropillar array structure. (**d**) The spacing of the micropillar array is 20.67 μm.

The PDMS microfluidic chip with the 3D focusing microfluidic channel and the micropillar array structure fabricated according to the above process was observed under scanning electron microscopy. Figure 9 shows that the structure of each part is complete, the channel is unobstructed, and the micropillar array and the 3D focusing channel largely meet the design requirements. 

### 4.2. Experimental Test Results

#### 4.2.1. Enrichment Test Results of the Chip

**Figure 10:**
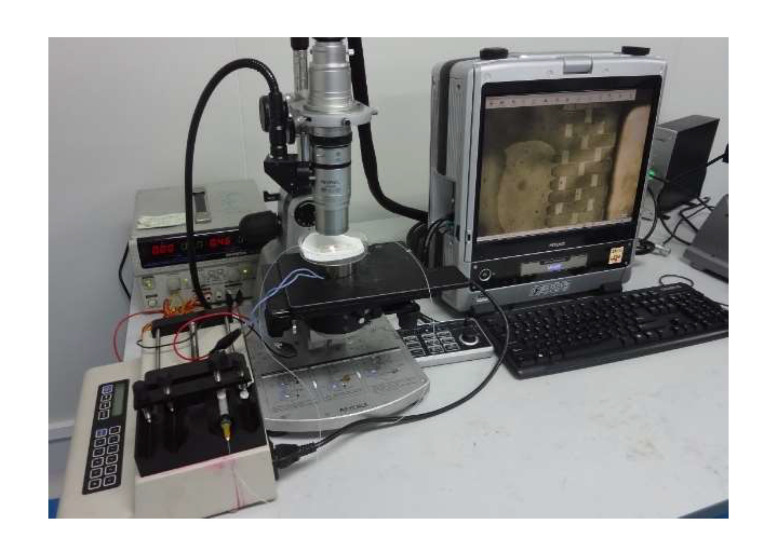
The experimental test device for the microfluidic chip.

**Figure 11:**
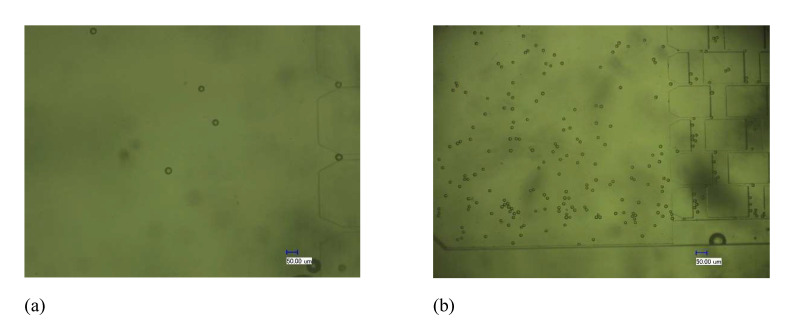

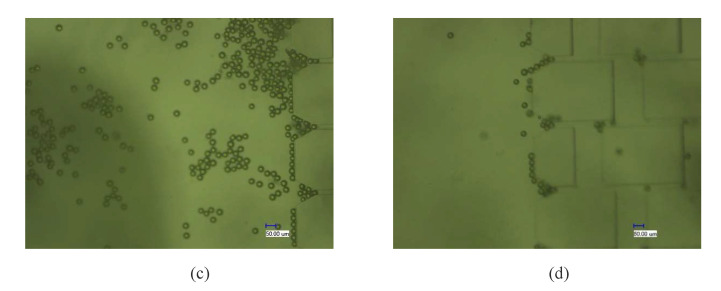
The changes in polyethylene particles enrichment under electron microscope. (**a**) The polyethylene particles enter the micropillar array when power is off. (**b**) The polyethylene particles flow over the micropillars when power is off. (**c**) The polyethylene particles are enriched at the moment when power is on. (**d**) The enriched polyethylene particles are washed away with buffer after power is off.

The fabricated PDMS substrate and PDMS cover sheet were bonded and packaged and were tested using the device in Figure 10. In the test, a DI water solution containing fluorescence micro particles with a diameter of 22 μm (monodispersed fluorescent polystyrene micro particles, manufactured by Tianjin BaseLine Chromatography Technology Development Center, a particle size of 22 μm, and a concentration of 2.5% *w*/*v*) was used as the sample solution (0.5 mL fluorescence micro particles solution dispersed into 100 mL of DI water), and DI water was used as the sheath solution and buffer solution. The sample injection rate of the microinjection pump was adjusted to 100 μL/min to slowly inject the sample solution into the microfluidic chip. The chip was observed under an electron microscope (Figure 11). When the electromagnetic device was not powered on, the polyethylene particles flew through the gap between the top of the micropillar array and the PDMS cover sheet. After turning on the power to generate the electromagnetic force, the PDMS cover sheet deformed and gradually blocked polyethylene particles in front of the micropillar array, causing an enrichment of polyethylene particles, while other solutions continued to pass freely. After turning off the power, the elastic deformation of the cover sheet disappeared, the gap between the micropillar array and the cover sheet was restored, and the enriched polyethylene particles gradually flowed through the gap with the buffer. 

#### 4.2.2. The Focusing and Counting Effect of the Chip

**Figure 12:**
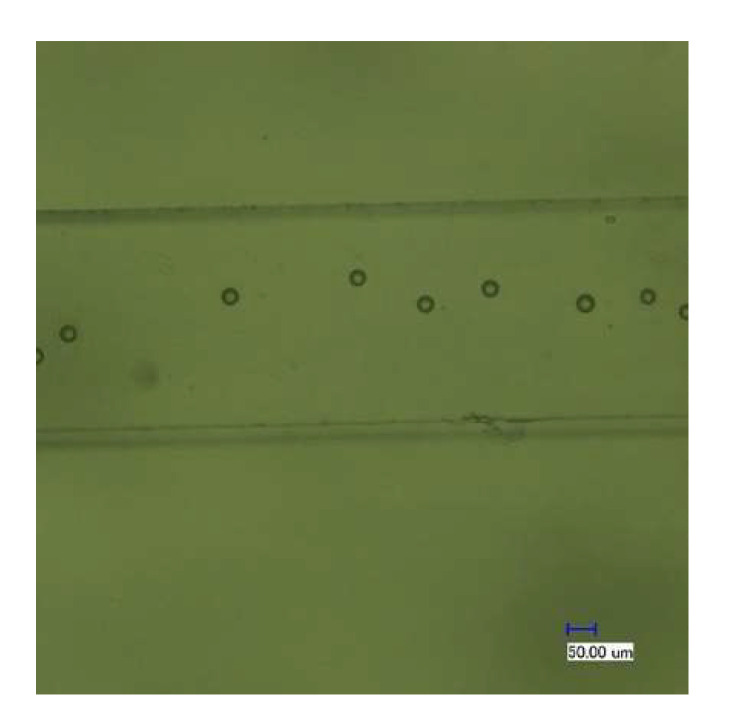
The polyethylene particles observed at the outlet of the microfluidic chip.

As described above, the polyethylene particles were enriched in the designed microfluidic chip. When the electromagnetic drive device was powered off, the microfluidic chip was no longer subjected to an external force, and the internal channel of the chip was reopened. At this time, DI water solution was injected to release the polyethylene particles that were enriched around the micropillar array, and at the same time sheath solution was injected at a certain rate through the sheath solution inlet. The DI water solution containing the polyethylene particles was used as the sample solution at a new concentration to pass through the 3D focusing channel. After the polyethylene particles were enclosed by the sheath solution on both sides, the polyethylene particles were focused and counted at the sample outlet. After multiple tests and considering the relationship between the sample focusing width and flow rate ratio mentioned in the literature [17], the injection rate was set to 100 μL/min when enriching polyethylene particles, and when releasing polyethylene particles, the buffer and sheath solution were injected at the same time at a flow-rate ratio of 1:4. Electron micrographs of polyethylene particles in the sample outlet channel showed that polyethylene particles passed through the channel one by one (Figure 12). 

## 5. Discussion

### 5.1. Optimization of the Chip Structure

**Figure 13:**
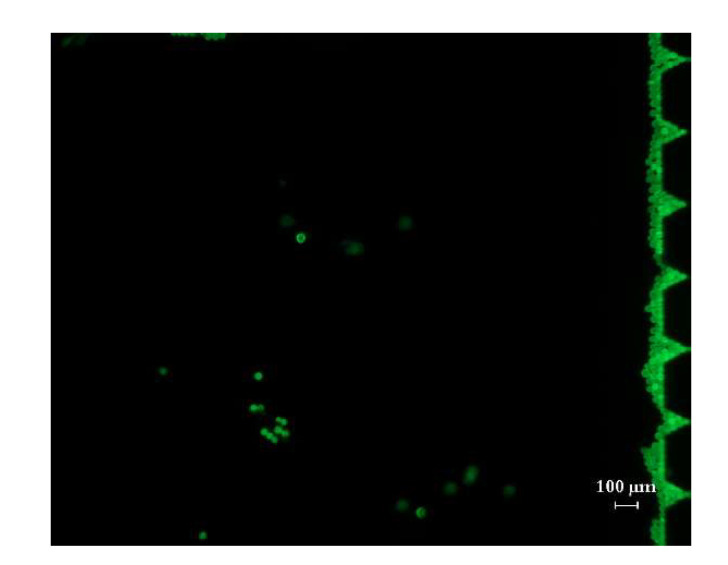
Fluorescence polystyrene micro particles are enriched in front of the first row of micropillars.

The chip in this study has only one micropillar array structure with spacing, which is only suitable for processing a small amount of sample. When the sample quantity injected is relatively large, all the particles to be tested could gather in front of the first row of the micropillar array after the power is on. As shown in Figure 13, the observation results under fluorescence microscopy showed with the increase in enrichment time, the enriched micro particles blocked the smooth flow in the chip, which may even damage the chip. However, as a possible application in the future, the chip may be used for the enrichment of a small number of tumor cells, because in the actual patient’s blood samples, the number of tumor cells is very small, and the blood cells can pass through the capture unit smoothly. This channel blocking effect will hardly occur in clinical samples. To improve the smoothness of sample processing, a micropillar array with spacing could be designed to have multiple sizes in the same array, and the size from the front row to the back row could gradually shrink to a size similar to the diameter of the micro particles. This design would help to improve the capture efficiency of the particles and their enrichment. 

### 5.2. The Effects of Flow Rate and Concentration on the Enrichment Efficiency of the Chip

**Figure 14:**
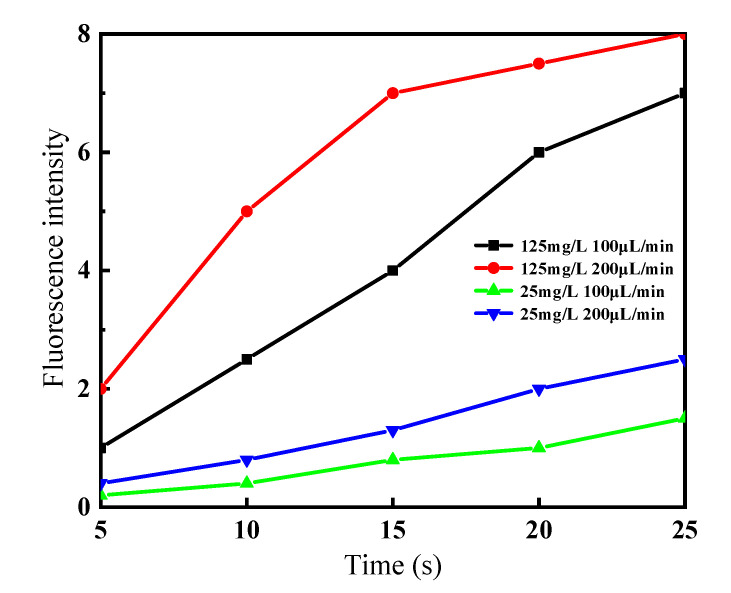
Comparison of fluorescence intensity of enriched polyethylene particles at different concentrations and different flow rates.

The tests found that the enrichment of polyethylene particles was affected by the flow rate and sample concentration. Figure 14 shows the fluorescence intensity of enriched polyethylene particles over time when polyethylene particles solutions of two different concentrations (one was 125 mg/L by dispersing 0.5 mL of polyethylene particles solution into 100 mL of DI water; the other was 25 mg/L by dispersing 0.1 mL of polyethylene particles solution into 100 mL of DI water) were injected into the chip at different rates of 100 and 200 μL/min, respectively. For the same concentration, the increase in flow rate enriched the fluorescent particles in a short time initially, but when the enrichment reached a certain degree, the enrichment rate decreased with time due to the pile-up of fluorescent particles in front of the micropillar array, and flow inside the channel was obstructed. In the experiment, when the sample solution was injected at a rate of 300 μL/min, after 30 s the chip was damaged. For the same flow rate, the enrichment rate of the low-concentration solution was low, and it did not cause much channel blockage, and the enrichment effect was maintained. 

## 6. Conclusions

In this study, a microfluidic chip that can be used for micro particles counting and detection was fabricated using a micro-electromechanical-systems (MEMS) process. The chip integrated a micropillar array for specific size micro particles enrichment and a 3D-focusing microfluidic channel for micro particles counting. The designed chip was tested using 22 μm polyethylene fluorescent micro particles, and results verified that the chip could realize the proposed enrichment, focusing and counting functions. As a detection chip, the PDMS mold has a low production cost, and the chip is disposable, which reduces cross-contamination of samples. If the chip is applied to CTCs detection in the future, external equipment used for CTCs detection, such as a laser light source, electromagnetic drive device, micropump, and computer, as active components can be affixed to the same platform, and this study can provide a practical method for low-cost and high-efficiency CTCs detection. Meanwhile the microfluidic chip fabrication method proposed in this study provides a strong theoretical and practical basis for the integrated structural design and micromachining process of other complex microstructures in the same field.
